# GABA signalling in human pancreatic islets

**DOI:** 10.3389/fendo.2023.1059110

**Published:** 2023-02-20

**Authors:** Zhe Jin, Sergiy V. Korol

**Affiliations:** Department of Medical Cell Biology, Uppsala University, Uppsala, Sweden

**Keywords:** GABA_A_ receptor, β cell, diabetes mellitus, insulin secretion, blood glucose, mixed-identity cell, T1D mouse model, GABA tolerance

## Abstract

The pancreatic islets are essential microorgans controlling the glucose level in the blood. The islets consist of different cell types which communicate with each other by means of auto- and paracrine interactions. One of the communication molecules produced by and released within the islets is γ-aminobutyric acid (GABA), a well-known inhibitor of neuronal excitability in the mammalian nervous system. Interestingly, GABA is also present in the blood in the nanomolar concentration range. Thus, GABA can affect not only islet function *per se* (e.g. hormone secretion) but also interactions between immune cells and the pancreatic islet cells in physiological conditions and in pathological states (particularly in type 1 diabetes). In the last decade the interest in GABA signalling in islets has increased. The broad research scope ranges from fundamental physiological studies at the molecular and cellular level to pathological implications and clinical trials. The aim of this mini-review is to outline the current status of the islet GABA field mostly in relation to human islets, to identify the gaps in the current knowledge and what clinical implications GABA signalling may have in islets.

## Introduction

Hormone secretion from pancreatic islet cells is tightly controlled by nutrients, neurotransmitters and other hormones in order to maintain a glucose homeostasis. Elements of different neurotransmitter signalling machineries such as choline- (1), glutamate- (2) or γ-aminobutyric acid (GABA)-ergic systems (3–7) are found in both animal and human pancreatic islet cells. Mechanism of action of such systems on islet cell function and particularly hormone secretion is not well understood. One of the least studied neurotransmitter systems in human pancreatic islets at molecular level is the GABAergic system. There are reports about beneficial effects of GABA on human islet function (8, 9) but underlying mechanisms are not fully understood.

The GABA signalling has been studied in detail in the central nervous system (CNS) where it plays a vital role in maintaining the balance between inhibition and excitation of neurons. The GABA inhibitory function in the CNS is mainly mediated by two classes of GABA receptors, type A and B (GABA_A_ and GABA_B_, respectively). GABA_A_ are ionotropic receptors assembled from five subunits forming heteropentameric chloride channel. The GABA_A_ receptor subunits include 19 isoforms in mammals: α1–6, β1–3, γ1–3, δ, ε, θ, π, ρ1–3. Synaptic and extrasynaptic GABA_A_ receptors mediate rapid inhibitory signals by means of fast postsynaptic membrane hyperpolarization and slower (tonic) inhibitory responses, respectively (10–13). GABA_B_ receptors are metabotropic G protein-coupled receptors, consisting of two subunits, GABA_B1_ and GABA_B2_. For normal function of GABA_B_ receptors both subunits should be assembled together (14, 15). GABA_B_ receptors provide slow inhibitory signals through G proteins and second messengers such as cyclic adenosine monophosphate (cAMP), inositol trisphosphate (IP_3_) or diacylglycerol (DAG) (15, 16). Interestingly, in human islets and human peripheral blood mononuclear cells (PBMCs) only GABA_B1_ mRNA transcript was detected in contrast to murine islet and immune cells where both subunits GABA_B1_ and GABA_B2_ were found (7, 17). However, it is possible to induce GABA_B2_ subunit in intact human islets under specific stimulation (18).

Proper function of the GABA signalling is important not only in the CNS but also for normal activity of pancreatic islets. Here we discuss the functional properties of the GABAergic system and how they are related to hormone secretion from islet cells under physiological and pathophysiological conditions with focus on human pancreatic islets. We summarize the current knowledge in the islet GABA field and what clinical implications it may have.

## Molecular aspects of GABA signalling in pancreatic islets

GABA, GABA_A_ receptor subunits and other components of the GABAergic system (e.g. glutamic acid decarboxylase, GAD) were detected at mRNA and protein levels in human islets in different studies by a variety of methods (5–7, 19–21). In 2010 it was reported that GABA is an autocrine transmitter in human β cells having excitatory effect and thus stimulating the insulin secretion (5). The levels of GABA_A_ receptor expression and correspondent Cl^–^ currents mediated by the receptors were compared in dispersed α, β and δ cells. β and δ cells demonstrated remarkable GABA-activated currents. GABA_A_ receptor-mediated currents in α cells were negligible. Interestingly, the GABA release from β cells was shown to be both, glucose-dependent and independent. Glucose-dependent GABA secretion was accompanied by the insulin co-release from large dense-core vesicles (LDCVs) that indicates co-localisation of GABA and insulin in the same vesicle. However, the mechanism of glucose-independent route of the GABA release was not identified (5). Almost ten years later this phenomenon was described in a study by Menegaz with co-authors (21). The authors found that the majority of GABA produced by β cell is present in the cytosol and released to the interstitium by volume regulatory anion channel (VRAC), encoded by LRRC8A (SWELL1), in a pulsatile manner that in turn drives oscillatory pattern of insulin secretion described before (22, 23). In addition, the GABA action on pulsatile insulin release was inhibitory and stabilizing in terms of periodicity of insulin pulses. The discrepancy between findings here and in (5) can be explained by differences in experimental conditions between the studies: continuous hormone secretion monitoring versus static incubation, differences in basal glucose concentrations, intact islets versus dispersed single cells or cell clusters and cells overexpressing selected GABA_A_ receptor subunits or islet preincubation with insulin. Nevertheless, these and other studies have shown that the main effect of GABA in islets is committed through the GABA_A_ receptors.

A very detailed analysis of functional properties of GABA_A_ receptors in human islet cells was performed recently (7). The data for single-channel GABA_A_ receptors from both types of donors, non-diabetic (ND) and type 2 diabetes (T2D), were analysed. Two subgroups of GABA_A_ receptors in both donor types were identified and designated islet GABA_A_ receptors group I (iGABA_A_RI) and II (iGABA_A_RII) with the cord conductance 37 and 76 pS, respectively. The properties of single-channel GABA_A_ receptors at room temperature (RT) and 34°C, the dependence of single-channel conductance on GABA concentration, and the modulation of channel activity by anaesthetics and anxiolytic pharmaceuticals were examined. The kinetic modelling revealed that in islets from T2D donors GABA_A_ receptors become more sensitive to GABA. We also found that the amplitudes of currents through and open probability of single-channel GABA_A_ receptors in α cells were significantly lower than in β cells (7). In δ cells, in turn, the amplitudes of GABA_A_ receptor-mediated currents were much larger than in the other two cell types, and these currents had transient and synaptic-like character. In general, distribution of GABA_A_ receptor-mediated currents in α, β and δ cells in our study (7) resembled that presented by Braun and colleagues (5) in spite of differences in types of currents recorded i.e. whole-cell single-channel vs. GABA puffer-induced whole-cell synaptic-like currents, tissue preparation i.e. intact islets vs. dispersed cells, and no insulin treatment (7) vs. preincubation with insulin (5).

The iGABA_A_Rs were selectively modulated by “classical neuronal” GABA_A_R drugs such as diazepam (benzodiazepine), propofol and pentobarbital (both anesthetics) reflecting significant increase in the mean single-channel current (I_mean_) and opening frequency of iGABA_A_Rs. In contrast, hypnotic zolpidem did not change these parameters. Since zolpidem binds with the highest affinity to α1 subunit containing GABA_A_Rs (24), insensitivity of iGABA_A_Rs to zolpidem is very likely due to low or no expression of α1 subunit in human β cell GABA_A_Rs. The incretin glucagon-like peptide-1 (GLP-1) at physiological concentration increased the frequency of iGABA_A_RI single-channel openings (7). We have previously shown in hippocampal CA3 pyramidal neurons that one of the mechanisms of action of GLP-1, and its analogue exendin-4, is a stimulation of GABA release from presynaptic vesicles (25, 26). Similarly, in pancreatic β cells GLP-1 receptor activation leads to increase of intracellular cyclic adenosine monophosphate (cAMP) and many secondary actions that eventually result in vesicular release of insulin (27). Thus, under GLP-1 application secretion of insulin from LDCVs is expected to be accompanied by GABA release from the same vesicles and potentially also from synaptic-like microvesicles (SLMVs) (4), elevating interstitial GABA that enhances iGABA_A_RI single-channel activity in auto- and paracrine manner. However, in order to accumulate GABA, SLMVs are expected to express the vesicular GABA transporter (VGAT) but according to the recent study (21) this is not the case. It was found that more than 99% of human β cells do not express VGAT. Vesicular GABA transporter was detected only in a small subpopulation of human β cells as well as in the δ cells, co-localising with GABA in vesicular compartments. Moreover, not all LDCVs contain GABA (5). Thus, it is possible that upon GLP-1 receptor activation in β cells, the local intracellular changes in osmolarity may occur due to cAMP-dependent reactions that lead to activation of SWELL1 channels and eventual release of GABA (28) that in turn, increases the frequency of iGABA_A_RI single-channel openings (7).

## Paracrine and autocrine function of GABA in human islets

Human islets have a more diffuse distribution of cells and the majority of β cells are closely associated with other endocrine cells (29). Therefore, GABA released from β cells may have both autocrine and paracrine effects in human islets. In the CNS GABA mediates inhibitory function in the majority of mature mammalian neurons. In human islets, the GABA autocrine effects on insulin secretion from β cells are more diverse and uncertain. According to several reports, in human as well as murine β cells GABA stimulated insulin secretion (5, 30). According to the recent study, GABA caused inhibition of insulin secretion and stabilized periodicity of insulin pulses in human β cells (21). In our hands, in some preparations GABA was inhibitory, in the others stimulatory in relation to the insulin release but, in both cases, these effects were reversed by the specific GABA_A_ receptor antagonist picrotoxin (7). In a study by Braun and colleagues, the selective blockade by another GABA_A_ receptor antagonist, SR95531 (gabazine), led to significant inhibition of insulin secretion only at 6 mM glucose but had no effect at lower (3 mM) or higher glucose concentrations (10 and 20 mM) (5). Similarly, the same antagonist SR95531 neither had the effect on insulin secretion at 1 nor 16.7 mM glucose in the study reported by Birnir’s team (6). However, the incubation with a GABA_B_R antagonist CPG55845 potentiated the insulin release in human islets in high glucose concentration (16.7 mM), suggesting GABA_B_R may mediate the inhibitory autocrine GABA effect (6). Variable effects of GABA on β cell insulin secretion at different glucose concentrations have been observed before, see e.g. (31). In case of GABA_A_R channels, the displacement from the chloride reversal potential (E_Cl_), but not the membrane potential value *per se*, defines the driving force and its direction, and thus the effects of GABA (32). When the glucose concentration increases, β cell depolarizes and membrane potential approaches E_Cl_. Thus, activating of GABA_A_Rs will not depolarize the cell anymore but rather, reduce excitability (and consequently insulin secretion) by clamping the membrane potential to E_Cl_.

GABA receptors are also detected in human α and δ cells. Blockage of GABA_A_Rs enhanced glucagon secretion in human islets in both low and high glucose concentrations (6, 33). Moreover, a recent clinical trial has shown that the combination of low dose GABA and GAD decreased fasting and meal-stimulated serum glucagon in newly diagnosed T1D patients (34). In contrast, inhibition of GABA_B_R did not affect glucagon release (6). These findings suggest that GABA has an inhibitory paracrine effect on glucagon secretion *via* GABA_A_Rs in human islets. The effect of GABA on somatostatin (SST) secretion is underexplored. Braun with co-authors (5) have shown that inhibition of GABA_A_Rs in human islets decreased SST secretion in both low (3 mM) and high (20 mM) glucose, indicating the stimulatory GABA effect on SST secretion (5). However, some preliminary data demonstrated the transient application of GABA (100 μM) increased SST secretion in low glucose but reduced SST in high glucose in human islets (35). In summary, the autocrine and paracrine actions of GABA in human islets appear to be complicated, depending on the glucose concentrations (low vs. high), insulin secretion assay (static vs. dynamic) and experimental design (*ex vivo* vs. *in vivo*).

## Single-cell transcriptomics and GABA signalling in islet cells expressing more than one hormone gene

Recently the pancreatic islet field advanced substantially with the development of a single-cell transcriptome technology (36–39). It allowed not only to see the genetic composition of every single cell of the tissue at a definite static time moment but also the observation of changes in the transcriptome of the cells after treatment (given that the treated subpopulation of cells is taken from the same population as a control group) as well as identification of genes as risk factors for different pathologies (e.g. type 2 diabetes, obesity, etc.) (38). Moreover, single-cell transcriptomics reveals the unique genetic signature for each cell type. Some studies even looked for correlations between cell genetic identity and functional manifestations, see e.g. (39). To be “pure” α, β or δ cell, the cell should express among other genes a single hormone transcript, *GLUCAGON* (*GCG*), *INSULIN* (*INS*) or *SOMATOSTATIN* (*SST*), respectively. Nevertheless, increasing number of studies are emerging reporting that among the major, “monohormonal” cell types in the endocrine pancreas, there are also cells expressing more than one hormone transcript (40–43). Various reasons for such co-expression have been suggested: different developmental stages of the major cell types (44, 45), stress (41) or changing conditions in the body (e.g. pregnancy, diabetes, obesity) (40, 46–48). The GABAergic system in islets not only modulates exocytosis (7), glucagon and insulin secretion (5, 6), but also regulates β cell proliferation (49, 50). It has also been reported that GABA signalling is involved in islet cell identity-change process, particularly α-to-β cell transdifferentiation (51–53), a process which however considered controversial (54, 55). The tentative explanations for the contradictory results could be differences in used animal models and their number, in lineage-tracing methods, and/or in diet and animal housing conditions (56). The bottom line is, however, the cells with mixed identity in terms of hormonal transcripts have been identified. Moreover, if to combine functional assay(s) with a single-cell content analysis it could be possible to characterize or predict the subtype of a mixed-identity cell. Recently we examined proportions of subpopulations of mono- and multihormonal cell types in intact pancreatic islets from ND and T2D donors (42). We found a decreasing of percentage of pure *INS*-expressing β cells in islets from T2D donors relative to the respective percentage in islets from ND individuals. At the same time, the percent of the mixed-identity cells containing *GCG* as well as of pure *GCG*-expressing α cells, increased in islets from T2D donors. Our results are in accordance with the studies showing a decrease in β cell mass and at the same time the expansion of the relative amount of α cells in islets of T2D donors if to compare with control subjects (57, 58). If this change in the distribution of such cells is a reason or a consequence of diabetes development is yet to be determined. After studying the patterns of activity of iGABA_A_R single-channels we also found a strong correlation between relative levels of expression of *GCG*, *INS* and *SST* and the frequency of iGABA_A_R single-channel openings in multihormonal mixed-identity cells. Particularly, in the cells we call α/β, the smaller value of *GCG*/*INS* ratio (at *GCG*/*INS* << 1, meaning that *INS* component is prevailing), the higher frequency of iGABA_A_R single-channel openings was recorded in mixed-identity cells. In contrast, the larger value of *GCG*/*INS* > 1, the lower frequency of iGABA_A_R single-channel currents and their amplitudes. Moreover, in the mixed-identity cells with mRNA expression ratio *GCG*/*INS* < 1, the iGABA_A_R single-channel currents were potentiated by GLP-1 (50 pM) application but were not affected in the cells with *GCG*/*INS* > 1 (42). Taken into account our previous functional characterization of iGABA_A_Rs in human islet cells (7), the cells with *GCG*/*INS* < 1 in terms of iGABA_A_R single-channel activity and sensitivity to GLP-1 behave similarly to β cells and were named “β-like cells”, and cells with *GCG*/*INS* > 1 were resembling the α cells and named α-like cells. Thus, the iGABA_A_R single-channel current characteristics can serve as functional markers for discrimination among different subpopulations of the mixed-identity pancreatic islet cells.

## Preclinical studies, clinical trials and potential clinical implications

**Figure 1:**
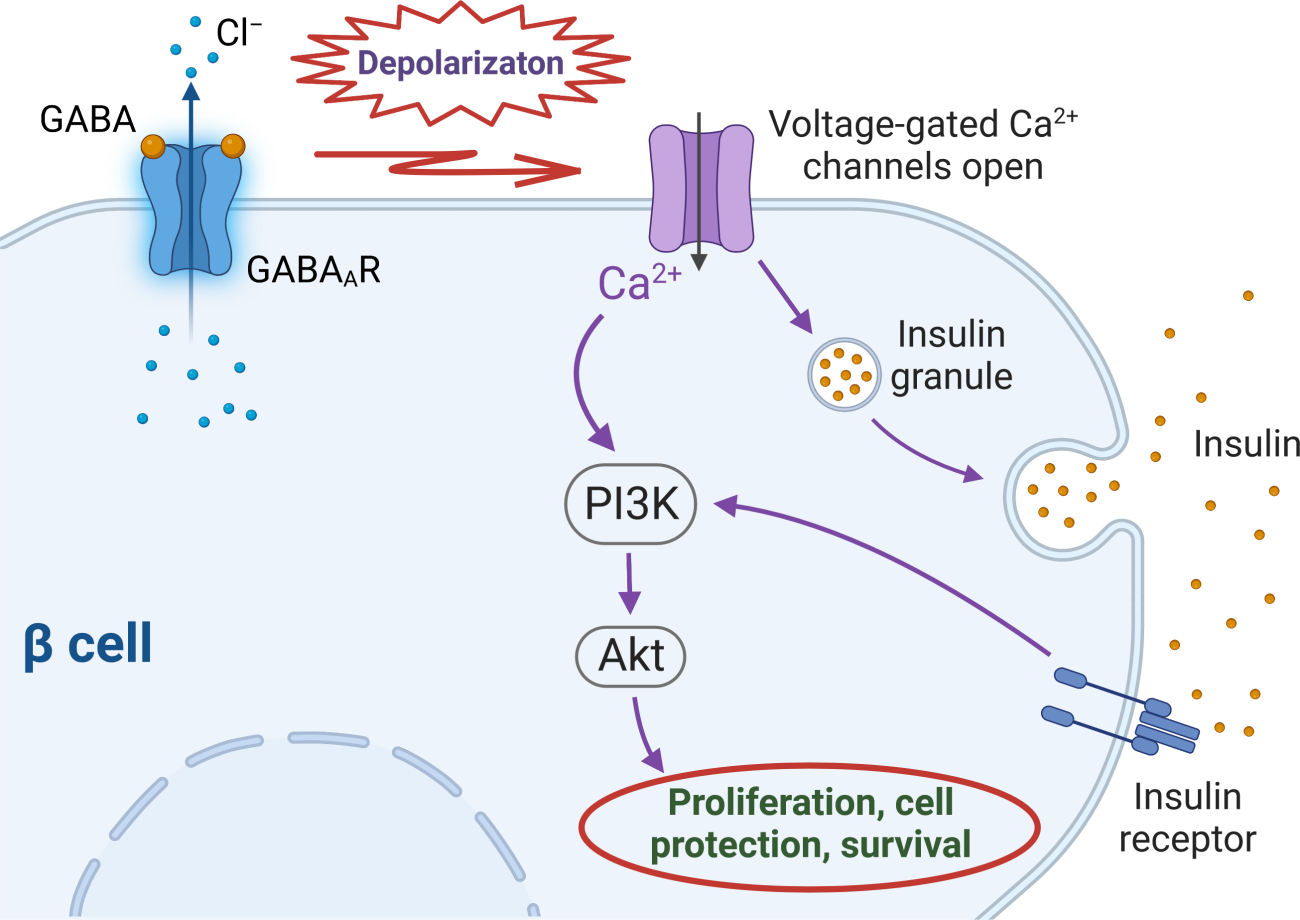
Intracellular cascades triggered by activation of GABA_A_ receptors in human β cells. Upon GABA binding to GABA_A_ receptors (GABA_A_Rs) chloride (Cl^–^) leaves the cell along the driving force created by the difference between the membrane potential and Cl^–^ equilibrium potential (E_Cl_), that depolarizes the cell membrane above the threshold of activation of voltage-gated calcium (Ca^2+^) channels (VGCCs). Ca^2+^ entrance through VGCCs stimulates insulin secretion which activates insulin receptor and subsequently – PI3K/Akt pathway. The influx of intracellular Ca^2+^ through VGCCs also activates PI3K/Akt cascade. Both routes eventually stimulate mechanisms of β cell proliferation and survival.

The importance of GABA in physiology of pancreatic islets is becoming more recognisable. There are number of studies reporting not only effects of GABA on islet hormone secretion (5, 6, 21) but also the trophic and regenerative (9, 50) properties of GABA on islet cells. Several preclinical studies with the use of animal models demonstrated not only regenerative effect of GABA on a β cell mass but also a concomitant suppressive action on immune cells (17, 30, 59–61). In particular, in the work by Soltani and colleagues (30) intraperitoneal GABA administration induced proliferation of β cells *in vivo*. Furthermore, in INS-1 cell line and in isolated CD1 mouse islets, GABA decreased the rate of apoptosis induced by streptozotocin (STZ) or inflammatory cytokine cocktail, respectively. In the other mouse model (MDSD) the authors also demonstrated that administration of GABA resulted in reducing circulating pro-inflammatory cytokines (IL-1Β, TNF-α, IFN-γ and others) and at the same time, the level of anti-inflammatory cytokine IL-10 was not changed. Overall, the study demonstrated that GABA exerts protective effect on β cells, possesses immunoregulatory capacity and is also capable of normalizing the blood glucose level in two type 1 diabetes (T1D) mouse models (MDSD and NOD mice), giving thus the basis for the conclusion that GABA was able to reverse T1D, at least in these two mouse models. In the other study, the 12-week feeding of high-fat and low-dose STZ-induced T2D mellitus mice with GABA-rich yogurt improved different parameters such as serum insulin level, blood urea nitrogen (BUN), islet morphology and others (62). In addition, GABA treatment and combination therapy have been shown to improve glucose tolerance and insulin sensitivity in rodent models of T2D (63, 64). The mechanism of the putative favourable effect of GABA signalling on β cell function is summarized in **Figure 1**.

The GABA-related studies were also done on human subjects. Particularly, pharmacokinetics and pharmacodynamics of GABA in healthy volunteers was investigated (65). Oral administration of GABA increased circulating levels of both hormones, insulin and glucagon, but the ratio insulin-to-glucagon was not altered that presumably accounts for constant blood glucose levels during 24 hours in this study. These results are in accordance to those obtained in few earlier studies showing good tolerance of GABA_B_ receptor agonist baclofen as well as GABA itself in amounts several grams per day and demonstrating similar effects of these compounds on insulin and glucagon secretion and plasma glucose concentration (66, 67).

The GABA effects are also studied in several clinical trials to investigate potential therapeutic effects in diabetes. GABA was found to be tolerated by subjects with long-standing (68) and newly diagnosed T1D (34); moreover, the drug induced counter-regulatory response of the respective hormones glucagon, adrenaline, growth hormone and cortisol (68). It is worth to note that the initial counter-regulatory response before the GABA administration was blunted (68). Similarly, a recent randomized trial in children with newly diagnosed T1D has shown the combination of GABA and GAD reduced fasting and meal-stimulated serum glucagon levels although no effect was observed on C-peptide levels (34). The study of blood samples from other cohorts of T1D subjects revealed negative correlation between GAD antibody and endogenous GABA levels in the plasma (69). The negative correlation with respect to GABA concentration was also revealed for circulating systemic levels of pro-inflammatory cytokines IL-1Β, IL-12 and IL-15. In contrast, the pro-inflammatory cytokine IL-36 and anti-inflammatory molecule IL-37 correlated positively to the circulating levels of GABA. In addition, the levels of circulating GABA in subjects with T1D and in healthy controls were similar (69) that is in line with our previous finding (17). The slight differences between these two studies may be explained by methods used (mass spectrometry vs. ELISA) and sizes of samples. Altogether, although the systemic GABA concentration is not substantially affected in T1D, the local GABA levels in pancreatic islets play an important role in harnessing resident immune cells, and decreasing of β cell mass in T1D apparently weakens the local control of immune response in the islets.

## Conclusions and perspectives

GABA signalling is of major importance in brain function from the start of CNS development, through normal development and aging as well as in psychiatric and neurological disorders, and has been studied in the CNS for more than seven decades. In contrast, the concept of GABA as one of the integral components of human pancreatic islet function is considerably younger. That GABA affects the islet hormone (both insulin and glucagon) secretion, stimulates β cell regeneration and is suggested as a promoter of α-to-β cell transdifferentiation, clearly identifies GABA as a significant molecule in the human pancreatic islets (51, 52, 56, 70).

**Figure 2:**
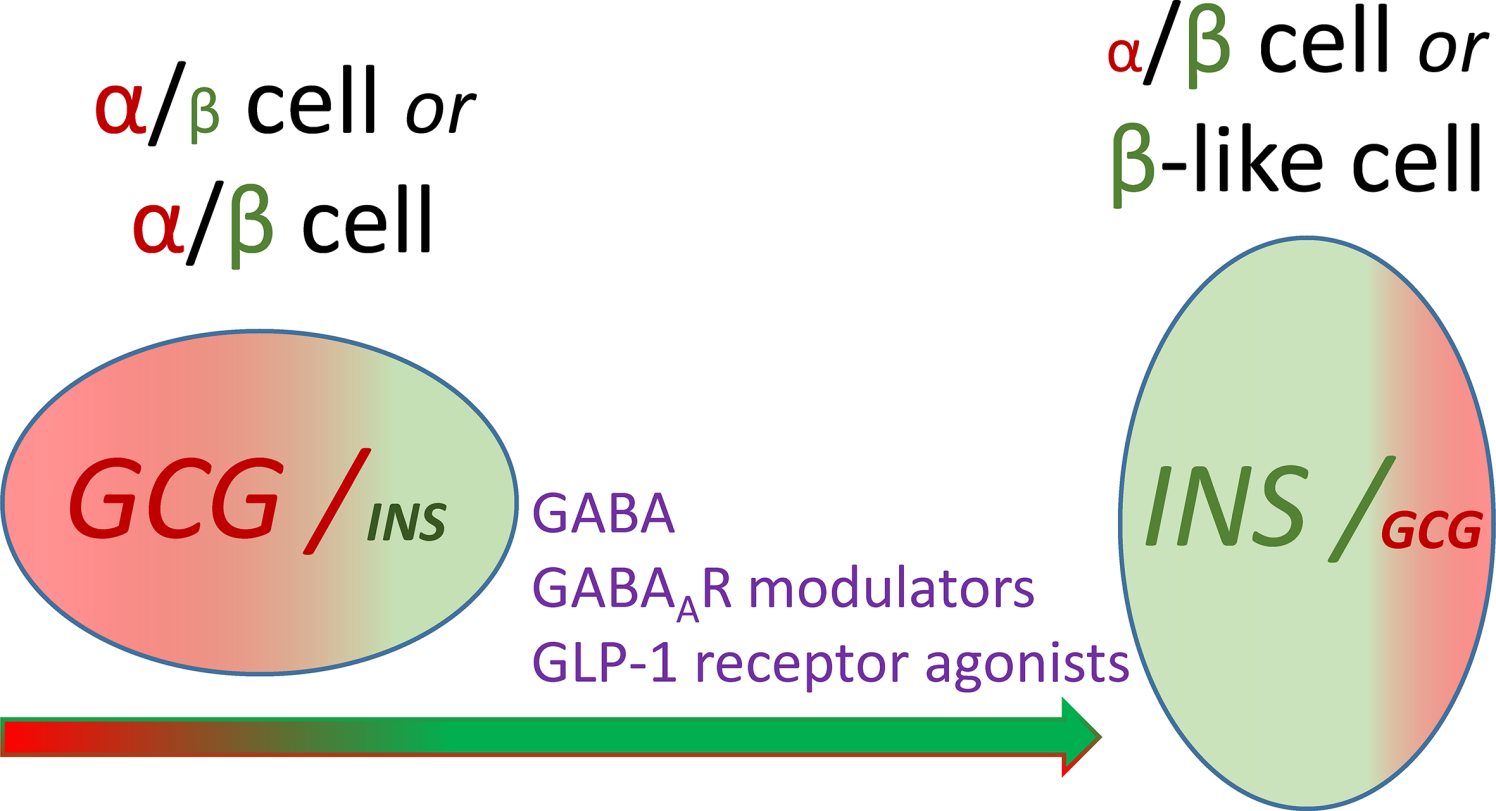
Hypothesis of controlled conversion of pancreatic cells with mixed identity. Conversion of the α/β cell with glucagon transcript (*GCG*) expression level higher than or similar to insulin transcript (*INS*) expression level, to the α/β cell with reciprocal expression pattern or to the cell with the β cell-like phenotype, respectively under the influence of molecules interacting with the GABA signalling system of the mixed-identity cell.

GABA demonstrated beneficial effects not only in rodent but also in human pancreatic islets (9, 30, 50, 70). Several studies showed that oral GABA administration is safe for human subjects (65, 68, 69) and activates GABA receptors only in peripheral tissues since it does not cross the blood-brain barrier at the concentrations used. Non-invasive strategies of treatment of diabetes mellitus are desirable. In light of this the question of the mixed-identity cells is especially relevant since the combination of GABA with other compounds (e.g. GABA signalling-modulating drugs [benzodiazepines, anesthetics]; GLP-1 receptor agonists [exendin-4, liraglutide, etc.]) may enhance the ability to change the identity of the cells diminished or altered by disease (**Figure 2**). However, further clinical trials using long-lasting GABA formulations, GABA analogs or combination therapy are required.

## Author contributions

Both authors contributed to writing the mini review. SVK created figures, edited and completed the final version of the manuscript. All authors contributed to the article and approved the submitted version.
